# Does the implementation of revised American College of Cardiology and American Heart Association (ACC/AHA) guidelines improve the identification of stillbirths and preterm births in hypertensive pregnancies: a population-based cohort study from South Asia and sub-Saharan Africa

**DOI:** 10.1186/s12884-024-06637-2

**Published:** 2024-06-29

**Authors:** Muhammad Imran Nisar, Ibrahim Kabole, Rasheda Khanam, Shahira Shahid, Bihila Abdalla Bakari, Nabidul Haque Chowdhury, Muhammad Farrukh Qazi, Arup Dutta, Sayedur Rahman, Javairia Khalid, Usha Dhingra, Tarik Hasan, Nadia Ansari, Saikat Deb, Dipak K. Mitra, Usma Mehmood, Fahad Aftab, Salahuddin Ahmed, Shahiryar Khan, Said Mohammad Ali, Saifuddin Ahmed, Alexander Manu, Sachiyo Yoshida, Rajiv Bahl, Abdullah H. Baqui, Sunil Sazawal, Fyezah Jehan

**Affiliations:** 1 Department of Pediatrics and Child Health, Aga Khan University, Stadium Road, Karachi, 74800 Karachi Pakistan; 2 Center for Public Health Kinetics, New Delhi, India; 3 Department of International Health, Johns Hopkins Bloomberg School of Public Health, Baltimore, MD USA; 4 Projahnmo Research Foundation, Dhaka, Bangladesh; 5 Department of Women’s and Children’s Health, Uppsala University, Uppsala, Sweden; 6 Department of Public Health, School of Health and Life Sciences, North South University, Dhaka, Bangladesh; 7 Public Health Laboratory-IDC, Pemba, Tanzania; 8 Department of Population, Family and Reproductive Health, Johns Hopkins Bloomberg School of Public Health, Baltimore, MD USA; 9 London School of Hygiene & Tropical Medicine, Faculty of Epidemiology and Public Health, London, UK; 10 Department for Maternal, Child, Adolescents and Ageing Health, World Health Organization, Avenue Appia 20, Geneva, 1211 Switzerland

**Keywords:** Hypertension, Pregnancy, Adverse outcomes, Pakistan, Tanzania, Bangladesh

## Abstract

**Background:**

Hypertensive disorders of pregnancy (HDP) are a significant cause of maternal mortality worldwide. The classification and treatment of hypertension in pregnancy remain debated. We aim to compare the effectiveness of the revised 2017 ACC/AHA blood pressure threshold in predicting adverse pregnancy outcomes.

**Methods:**

We conducted a secondary data analysis of the Alliance for Maternal and Newborn Health Improvement (AMANHI) biorepository study, including 10,001 pregnant women from Bangladesh, Pakistan, and Tanzania. Blood pressure was measured using validated devices at different antenatal care visits. The blood pressure readings were categorized as: normal blood pressure (systolic blood pressure (sBP) < 120 mm Hg and diastolic blood pressure (dBP) < 80 mm Hg), elevated blood pressure (sBP 120–129 and dBP < 80), stage 1 hypertension (sBP 130–139 or dBP 80–89, or both), and stage 2 hypertension (sBP ≥ 140 or dBP ≥ 90, or both). We estimated risk ratios for stillbirths and preterm births, as well as diagnostic test properties of both the pre-existing JNC7 (≥ 140/90) and revised ACC/AHA (≥ 130/80) thresholds using normal blood pressure as reference group.

**Results:**

From May 2014 to June 2018, blood pressure readings were available for 9,448 women (2,894 in Bangladesh, 2,303 in Pakistan, and 4,251 in Tanzania). We observed normal blood pressure in 70%, elevated blood pressure in 12.4%, stage 1 hypertension in 15.2%, and stage 2 hypertension in 2.5% of the pregnant women respectively. Out of these, 310 stillbirths and 9,109 live births were recorded, with 887 preterm births. Using the ACC/AHA criteria, the stage 1 hypertension cut-off revealed 15.3% additional hypertension diagnoses as compared to JNC7 criteria. ACC/AHA defined hypertension was significantly associated with stillbirths (RR 1.8, 95% CI 1.4, 2.3). The JNC 7 hypertension cut-off of ≥ 140/90 was significantly associated with a higher risk of preterm births (RR 1.6, 95% CI 1.2, 2.2) and stillbirths (RR 3.6, 95% CI 2.5, 5.3). Both criteria demonstrated low sensitivities (8.4 for JNC-7 and 28.1 for ACC/AHA) and positive predictive values (11.0 for JNC7 and 5.2 for ACC/AHA) in predicting adverse outcomes.

**Conclusion:**

The ACC/AHA criteria (≥ 130/80) identified additional cases of hypertension but had limited predictive accuracy for stillbirths and preterm births, highlighting the ongoing need for improved criteria in managing pregnancy-related hypertension.

**Supplementary Information:**

The online version contains supplementary material available at 10.1186/s12884-024-06637-2.

## Background

Hypertensive disorders of pregnancy (HDP) affect approximately 15% of women and are the second leading cause of maternal mortality worldwide [1]. In Low-and-Middle Income Countries (LMICs), the incidence of HDP is estimated to be 3.84 (3.16 to 4.62) million per 100,000 population in South Asia and 3.63 (3.02–4.33) million in sub-Saharan Africa [1]. HDP includes chronic hypertension, gestational hypertension, preeclampsia, and chronic hypertension superimposed with preeclampsia [2]. Genetic factors, pre-existing maternal comorbidities, and reproductive history increase the risk of HDP. It is characterized by abnormal placental development due to endothelial dysfunction and spiral artery remodeling during 8 to 12 weeks and 18 to 22 weeks of gestation. This impaired utero-placental perfusion and oxidative stress results in increased circulation of antiangiogenic factors such as tyrosine kinase 1, and a decrease of proangiogenic factors such as placental growth factor and vascular endothelial growth factor, leading to hypertension and glomerulopathy [3]. Elevated blood pressures throughout pregnancy are associated with an increased risk of maternal and fetal complications such as intrauterine growth restriction, small for gestational age and low birth weight, placental abruption, preterm birth, stillbirths, and cesarean delivery [4–6]. Currently, there is no clear consensus regarding the optimal BP threshold in pregnancy to initiate preventive measures and the target BP to achieve [2]. Thus, understanding the relationship between blood pressure thresholds and adverse outcomes attributable to HDP is crucial particularly in LMICs where the burden of hypertension-related morbidity is the greatest.

Previous studies in the non-pregnant population have shown that blood pressures even below hypertensive thresholds elevate cardiovascular risk, suggesting a need for preventive interventions [7]. In light of this evidence, in 2017 the ACC/AHA lowered its thresholds for Stage I and Stage II hypertension to reduce the lifetime risk of cardiovascular disease [8]. However, the American College of Obstetricians and Gynecologists (ACOG) has maintained its single diagnostic threshold for hypertension in pregnancy at a systolic blood pressure (SBP) ≥ 140 mm Hg or diastolic blood pressure (DBP) ≥ 90 mm Hg after 20 weeks of gestational age. The implications of the revised 2017 ACC/AHA clinical guidelines on the risk of adverse pregnancy outcomes remain unclear.

The Alliance for Maternal and Child Health Improvement (AMANHI) biorepository study enrolled 10,001 pregnant women between 8 to < 20 weeks of gestational age from Bangladesh, Pakistan, and Tanzania from May 2014 till June 2018 [9].

In this study, we compare the JNC7 with ACC/AHA defined hypertension cut-offs to predict adverse outcomes such as stillbirths and preterm births in the AMANHI cohort.

## Methods

### Study design and setting

We performed a secondary data analysis on a large cohort of pregnant women enrolled as part of the Alliance for Maternal and Child Health Improvement (AMANHI) biorepository study. Between May 2014 and June 2018, the AMANHI study enrolled 10,001 pregnant women between 8 to < 20 weeks of gestational age from Bangladesh, Pakistan, and Tanzania. Women were enrolled after confirmation of pregnancy and gestational age through ultrasound, at the time of enrollment, 24–28 or 32–36 weeks of gestation, at the time of birth and 6 weeks after delivery. At each contact, trained field workers collected detailed information on the health and care-seeking behavior of the pregnant woman using standardized tools across all sites [9]. A detailed description of the study sites and characteristics of the cohort has been published previously [9].

Trained community health workers (CHWs) measured women’s blood pressure and performed dipstick urinalysis for proteinuria at 24–28 weeks, 32–36 weeks, and 38–40 weeks, whereas postnatal blood pressure was checked at the 0–6-day visit and finally at 42–60 days of delivery. Blood pressure was measured using an automated pregnancy-validated oscillometric device (WatchBP® Home Monitor, Microlife®, Taipei, Taiwan). Women were instructed to sit quietly and rest for 15 min with their legs uncrossed. After the resting period, their measurements were taken with the participant sitting upright with proper back support and arm supported on a table or a surface at heart level. Blood pressure was then measured three times, with 3-min intervals between each reading. The blood pressure for the visit was calculated as the average of the second and third readings. All readings were stored electronically in.NET databases.

In cases where the blood pressure reading was greater than or equal to 140/90 mm Hg at least four hours apart during any of the visits, CHWs referred the pregnant woman to the study physician for further investigation. They were prescribed oral methyldopa (250 mg) and advised to follow up after a week. If blood pressure was recorded as greater than 150/100 mm Hg, the physician provided oral methyldopa (500 mg) and referred the women to a tertiary care hospital for further management. In cases where preeclampsia was suspected, they were referred to a comprehensive emergency obstetric care facility as advised by the study physician.

### Statistical analysis

The primary exposure variable was antenatal blood pressure measurements for each woman at each visit classified on the basis of the American College of Cardiology and American Heart Association (ACC/AHA) criteria as follows: normal blood pressure (sBP < 120 mm Hg and dBP < 80 mm Hg), elevated blood pressure (sBP 120–129 mm Hg and dBP < 80 mm Hg), stage 1 hypertension (sBP 130–139 mm Hg or dBP 80–89 mm Hg, or both), and stage 2 hypertension (sBP ≥ 140 mm Hg or dBP ≥ 90 mm Hg, or both). Blood pressure readings were also classified according to the Joint National Committee defined cut-off of (sBP ≥ 140 mm Hg or dBP ≥ 90 mm Hg, or both) which was used as a comparison group. For the primary analyses, we classified women according to the maximum blood pressure category reached across all visits. Women who were hypertensive before 20 weeks of gestation were regarded as having chronic hypertension, while those who were hypertensive at 20 weeks of gestation or later were regarded as having gestational hypertension or preeclampsia. Antepartum hemorrhage was defined as bleeding from or into the genital tract, occurring from 24 + 0 weeks of pregnancy and prior to birth. For the outcomes of interest, stillbirths were defined as babies who were born dead after 22 weeks of gestation. Among livebirths, preterm births were defined as livebirths before 37 weeks of gestation.

For descriptive purposes, all quantitative data were expressed as mean ± SD, and qualitative data as frequencies with percentages. Logistic regression was used to estimate risk ratios for the JNC 7 and ACC/AHA cut-offs with the two predefined outcomes as compared to normotensive women. The diagnostic test properties of blood pressure categories were assessed using sensitivity, specificity, number needed to diagnose (NND) and positive and negative predictive values. Confidence Intervals were calculated by standard methods. For each calculation, women with blood pressure equal to or higher than the given threshold were compared to those with blood pressure lower than the threshold. We excluded women with missing BP readings at enrollment, multiple gestations, more than one pregnancy during the study period, missing outcome information from the analysis. All analyses was performed using Stata version 17.0.

### Ethical statement

The AMANHI study received ethical approval from the local and institutional ethics committees of all the three sites. These included Zanzibar Health Research Ethics Committee (formerly ZAMREC) (ZAMREC/0002/OCTOBER/013) for Tanzania, ICDDR, B (PR12073) and John Hopkins University (IRB 00004508) for Bangladesh and Aga Khan University (2790-paeds-ERC-13) for Pakistan. In addition, the protocols for the biorepository study were also approved by the WHO Ethics Review Committee (RPC 532) and continuing approvals were sought yearly. Written informed consent was obtained from study participants in which all sample handling and study procedures were explained in detail.

## Results

**Fig. 1:**
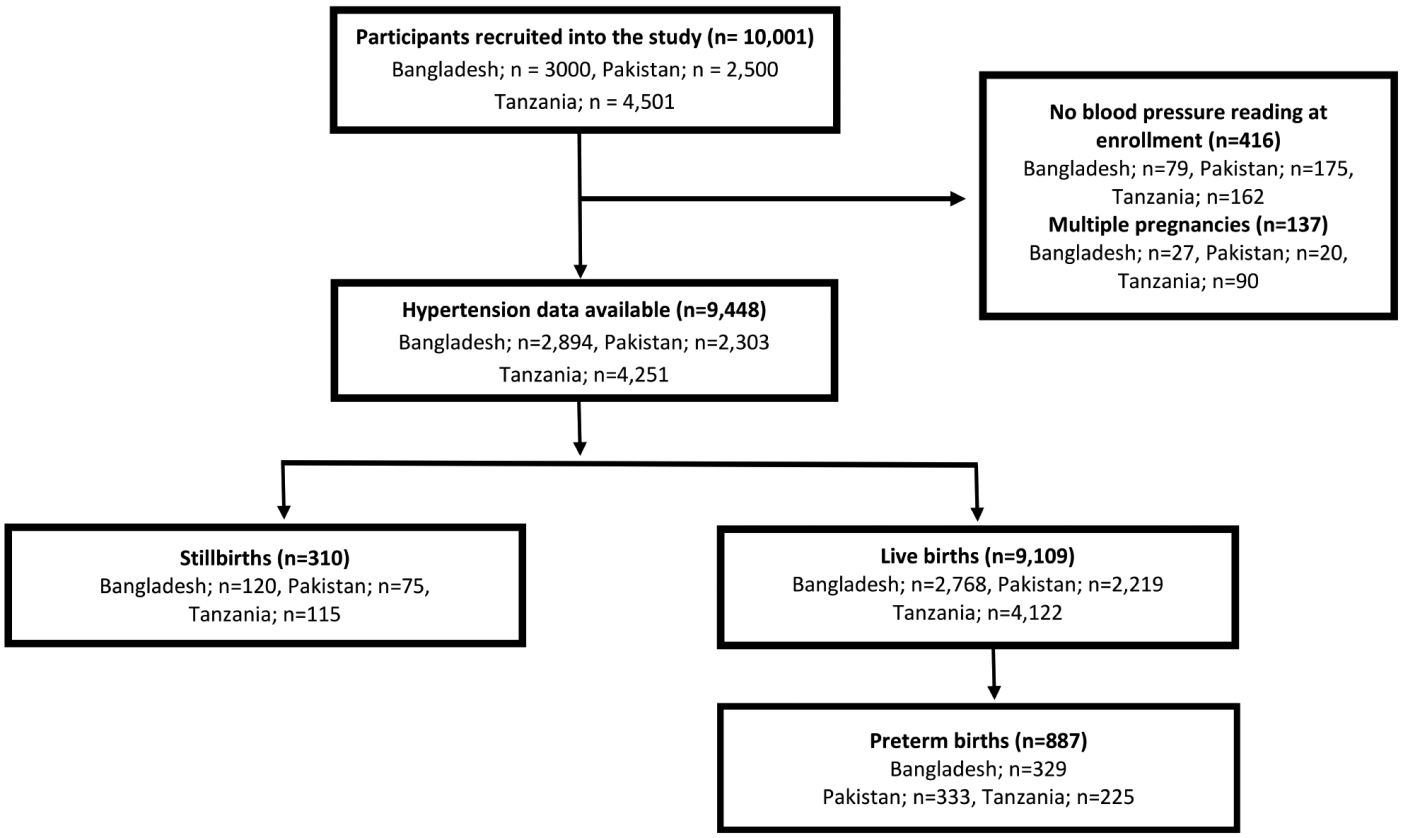
Flow of participants in the study

We enrolled 10,001 pregnant women in the AMANHI cohort, 3,000 in Bangladesh, 2,500 in Pakistan, and 4,501 in Tanzania. Blood pressure readings were available for 9,448 women. Throughout pregnancy, about two-thirds (70.0%) of the women had normal blood pressure, 1,168 (12.4%) had elevated blood pressure, 1,437 (15.2%) had stage 1 hypertension, and 234 (2.5%) had stage 2 hypertension respectively (Tables 1 and S1). The ACC/AHA cut-off diagnosed an additional 1,437 (15.2%) out of 9,448 women with hypertension as compared to the JNC7 cut-off. A majority of the pregnant women were between 20 and 29 years of age (51.9%), had received education up to the primary level (59.9%), and had 1–2 prior births (36.1%). We recorded 310 stillbirths and 9,109 livebirths, with most women delivering at term, but 887 delivering preterm (Fig. 1). In Bangladesh, 19.7% of the women had a mid-upper arm circumference cut-off of < 21, indicating severe undernutrition, while in Pakistan and Tanzania, the percentages were 11.5% and 2.4%, respectively. Approximately 10.8% of the women reported using tobacco (smoking and chewable) during pregnancy, while 5.1% and 5.8% reported antepartum infection and hemorrhage, respectively.

**Table 1:** Sociodemographic and clinical characteristics of the study participants

Characteristics	Bangladesh	Karachi	Pemba	Total
	*N* = 2,973	*N* = 2,478	*N* = 4,413	*N* = 9,864
	n (%)	n (%)	n (%)	n (%)
Maximal hypertension status ℇ	*n* = 2,894	*n* = 2,303	*n* = 4,251	*n* = 9,448
Normal BP	2,595 (89.7%)	1,635 (71.0%)	2,379 (56.0%)	6,609 (70.0%)
Elevated BP	72 (2.5%)	227 (9.9%)	869 (20.4%)	1,168 (12.4%)
Stage 1 hypertension	208 (7.2%)	394 (17.1%)	835 (19.6%)	1,437 (15.2%)
Stage 2 hypertension	19 (0.7%)	47 (2.0%)	168 (4.0%)	234 (2.5%)
Maternal age, years	*n* = 2,973	*n* = 2,478	*n* = 4,397	*n* = 9,848
<20 Years	1,047 (35.2%)	353 (14.2%)	561 (12.8%)	1,961 (19.9%)
20–29 Years	1,573 (52.9%)	1,422 (57.4%)	2,117 (48.1%)	5,112 (51.9%)
≥30 years	353 (11.9%)	703 (28.4%)	1,719 (39.1%)	2,775 (28.2%)
Maternal education, years	*n* = 2,964	*n* = 2,478	*n* = 4,397	*n* = 9,839
No Formal Education	189 (6.4%)	1,295 (52.3%)	0 (0.0%)	1,484 (15.1%)
Primary	1,073 (36.2%)	421 (17.0%)	4,397 (100.0%)	5,891 (59.9%)
Above primary	1,702 (57.4%)	762 (30.8%)	0 (0.0%)	2,464 (25.0%)
MUAC, cm^*^	*n* = 1,968	*n* = 1,943	*n* = 3,375	*n* = 7,286
Severely undernourished < 21	387 (19.7%)	223 (11.5%)	81 (2.4%)	691 (9.5%)
Mild to moderately undernourished ≥ 21 & < 23	667 (33.9%)	376 (19.4%)	306 (9.1%)	1,349 (18.5%)
Normal ≥ 23	914 (46.4%)	1,344 (69.2%)	2,988 (88.5%)	5,246 (72.0%)
Parity	*n* = 2,969	*n* = 2,478	*n* = 4,397	*n* = 9,844
No Previous births	1,122 (37.8%)	628 (25.3%)	865 (19.7%)	2,615 (26.6%)
1–2 Births	1,282 (43.2%)	1,085 (43.8%)	1,184 (26.9%)	3,551 (36.1%)
3–5 Births	515 (17.3%)	597 (24.1%)	1,505 (34.2%)	2,617 (26.6%)
>5 Births	50 (1.7%)	168 (6.8%)	843 (19.2%)	1,061 (10.8%)
	*n* = 2,965	*n* = 2,478	*n* = 4,375	*n* = 9,818
Improved toilet facility €	2,939 (99.1%)	2,473 (99.8%)	3,241 (74.1%)	8,653 (88.1%)
	*n* = 2,971	*n* = 2,478	*n* = 4,163	*n* = 9,612
Any tobacco use ¥	504 (17.0%)	500 (20.2%)	32 (0.8%)	1,036 (10.8%)
	*n* = 2,943	*n* = 2,423	*n* = 4,329	*n* = 9,695
Antepartum infection	41 (1.4%)	288 (11.9%)	168 (3.9%)	497 (5.1%)
	*n* = 2,750	*n* = 2,311	*n* = 3,952	*n* = 9,013
Antepartum hemorrhage	92 (3.3%)	293 (12.7%)	139 (3.5%)	524 (5.8%)

Note:

^*^ Source: Food and Nutrition Technical Assistance 2016, Use of Cutoffs for Mid-Upper Arm Circumference (MUAC) as an Indicator or Predictor of Nutritional and Health- Related Outcomes in Adolescents and Adults: A Systematic Review

¥ Any tobacco use includes sniffing, chewing, and smoking

ℇ Maximal Hypertension Ranges

Normal BP (sBP < 120 mm Hg and dBP < 80 mm Hg)

Elevated BP (sBP 120–129 mm Hg and dBP < 80 mm Hg)

Stage 1 hypertension (sBP 130–139 mm Hg or dBP 80–89 mm Hg, or both)

Stage 2 hypertension (sBP > = 140 mm Hg or > = dBP 90 mm Hg, or both)

€ Improved Toilet facility (Flush or pour flush toilet and Pit latrine)

Women who were diagnosed with hypertension as per ACC/AHA criteria had 23 more stillbirths per 1,000 women screened. Women with hypertension as per JNC7 criteria had 81 more stillbirths and 55 more preterm births per 1,000 women screened during the study (Tables 2 and 3). Using the JNC7 criteria for hypertension diagnosis, we found that the risk of preterm birth was 60% higher among hypertensive women than those with normal blood pressure (3.5%, RR 1.6, 95% CI 1.1, 2.2). However, when using the ACC/AHA criteria, there was no difference in the risk of preterm birth between hypertensive and normal women (17.3%, RR 1.0, 95% CI 0.8, 1.2). The JNC7 defined hypertension indicated a higher risk of stillbirth (11.2%, RR 3.6, 95% CI 2.5, 5.3) compared to that of ACC/AHA criteria (5.2%, RR 1.8, 95% CI 1.4, 2.3). Using JNC7 criteria, the number needed to diagnose one stillbirth was 12 and one preterm birth was 18, whereas with ACC/AHA criteria, it was 43 for stillbirth and 7154 for preterm birth respectively.

**Table 2:** Percentage difference between JNC7 and ACC/AHA blood pressure thresholds for preterm births including risk difference and number needed to diagnose

Blood pressure thresholds	Preterm Births *n* (%)	Term Births *n* (%)	RR (CI 95%)	Risk Difference	Number Needed to Diagnose
Overall	*n* = 887	*n* = 8,100			
JNC7					
Yes	31 (15.3%)	172 (84.7%)	1.6 (1.1, 2.2)	0.0552	18
No	856 (9.7%)	7,928 (90.3%)	Ref		
ACC/AHA					
Yes	153 (9.9%)	1,399 (90.1%)	1 (0.8, 1.2)	0.0001	7154
No	734 (9.9%)	6,701 (90.1%)	Ref		
Bangladesh	*n* = 329	*n* = 2,434			
JNC7					
Yes	2 (16.7%)	10 (83.3%)	1.4 (0.4, 5)	0.0478	21
No	327 (11.9%)	2,424(88.1%)	Ref		
ACC/AHA					
Yes	22 (10.5%)	187 (89.5%)	0.9 (0.6, 1.3)	0.0149	67
No	307 (12.0%)	2,247 (88.0%)	Ref		
Pakistan	*n* = 333	*n* = 1,874			
JNC7					
Yes	7 (17.5%)	33 (82.5%)	1.2 (0.6, 2.3)	0.0245	41
No	326 (15.0%)	1,841 (85.0%)	Ref		
ACC/AHA					
Yes	63 (15.1%)	353 (84.9%)	1 (0.8, 1.3)	0.0006	1452
No	270 (15.1%)	1,521 (84.9%)	Ref		
Tanzania	*n* = 225	*n* = 3,792			
JNC7					
Yes	22 (14.6%)	129 (85.4%)	2.8 (1.8, 4.2)	0.0931	11
No	203 (5.3%)	3,663 (94.7%)	Ref		
ACC/AHA					
Yes	68 (7.3%)	859 (92.7%)	1.4 (1.1, 1.9)	0.0225	44
No	157 (5.1%)	2,933 (94.9%)	Ref		

**Table 3:** Percentage difference between JNC7 and ACC/AHA blood pressure thresholds for stillbirths including risk difference and number needed to diagnose

Blood pressure thresholds	Stillbirths *n* (%)	Livebirths *n* (%)	RR (CI 95%)	Risk Difference	Number Needed to Diagnose
Overall	*n* = 310	*n* = 9,109			
JNC7					
Yes	26 (11.2%)	207 (88.8%)	3.6 (2.5, 5.3)	0.0806	12
No	284 (3.1%)	8,902 (96.9%)	Ref		
ACC/AHA					
Yes	87 (5.2%)	1,578 (94.8%)	1.8 (1.4, 2.3)	0.0234	43
No	223 (2.9%)	7,531 (97.1%)	Ref		
Bangladesh	*n* = 120	*n* = 2,768			
JNC7					
Yes	6 (33.3%)	12 (66.7%)	8.4 (4.3, 16.5)	0.2936	3
No	114 (4.0%)	2,756 (96.0%)	Ref		
ACC/AHA					
Yes	16 (7.1%)	209 (92.9%)	1.8 (1.1, 3)	0.0320	31
No	104 (3.9%)	2,559 (96.1%)	Ref		
Pakistan	*n* = 75	*n* = 2,219			
JNC7					
Yes	7 (14.9%)	40 (85.1%)	4.9 (2.4, 10.1)	0.1186	8
No	68 (3.0%)	2,179 (97.0%)	Ref		
ACC/AHA					
Yes	22 (5.0%)	418 (95.0%)	1.7 (1.1, 2.8)	0.0214	47
No	53 (2.9%)	1,801 (97.1%)	Ref		
Tanzania	*n* = 115	*n* = 4,122			
JNC7					
Yes	13 (7.7%)	155 (92.3%)	3.1 (1.8, 5.4)	0.0523	19
No	102 (2.5%)	3,967 (97.5%)	Ref		
ACC/AHA					
Yes	49 (4.9%)	951 (95.1%)	2.4 (1.7, 3.5)	0.0286	35
No	66 (2.0%)	3,171 (98.0%)	Ref		

The ACC/AHA and JNC7 blood pressure thresholds demonstrated low sensitivity as diagnostic markers for preterm birth (3.5 and 17.2, respectively), but high specificity (ACC/AHA 97.9 and JNC7 82.7, respectively). The positive predictive value was 15.3 for JNC7 and 9.9 for ACC/AHA, while the negative predictive value was 90.3 and 90.1 respectively. As diagnostic markers for stillbirth, both JNC7 and ACC/AHA blood pressure thresholds showed low sensitivity (8.4 and 28.1, respectively) but high specificity (97.7 and 82.7, respectively). The positive predictive value was 11.0 for JNC7 and 5.2 for ACC/AHA, while the negative predictive value was 97.0 for both thresholds (Table 4).

**Table 4:** Diagnostic accuracy of the BP thresholds in predicting preterm births and stillbirths

Blood pressure thresholds	Sensitivity	Specificity	Positive predictive value	Negative predictive value
Preterm births				
Overall				
JNC7	3.5(2.4, 4.9)	97.9(97.5, 98.2)	15.3(10.6, 21)	90.3(89.6, 90.9)
ACC/AHA	17.2(14.8, 19.9)	82.7(81.9, 83.5)	9.9(8.4, 11.5)	90.1(89.4, 90.8)
Bangladesh				
JNC7	0.6(0.1, 2.2)	99.6(99.2, 99.8)	16.7(2.1, 48.4)	88.1(86.8, 89.3)
ACC/AHA	6.7(4.2, 10)	92.3(91.2, 93.3)	10.5(6.7, 15.5)	88(86.7, 89.2)
Pakistan				
JNC7	2.1(0.8, 4.3)	98.2(97.5, 98.8)	17.5(7.3, 32.8)	85(83.4, 86.4)
ACC/AHA	18.9(14.9, 23.5)	81.2(79.3, 82.9)	15.1(11.8, 19)	84.9(83.2, 86.6)
Tanzania				
JNC7	9.8(6.2, 14.4)	96.6(96.0, 97.2)	14.6(9.4, 21.2)	94.7(94.0, 95.4)
ACC/AHA	30.2(24.3, 36.7)	77.3(76,0.0 78.7)	7.3(5.7, 9.2)	94.9(94.1, 95.7)
Stillbirths				
Overall				
JNC7	8.4(5.6, 12)	97.7(97.4, 98)	11.2(7.4, 15.9)	96.9(96.5, 97.3)
ACC/AHA	28.1(23.1, 33.4)	82.7(81.9, 83.4)	5.2(4.2, 6.4)	97.1(96.7, 97.5)
Bangladesh				
JNC7	5(1.9, 10.6)	99.6(99.2, 99.8)	33.3(13.3, 59)	96(95.2, 96.7)
ACC/AHA	13.3(7.8, 20.7)	92.4(91.4, 93.4)	7.1(4.1, 11.3)	96.1(95.3, 96.8)
Pakistan				
JNC7	9.3(3.8, 18.3)	98.2(97.6, 98.7)	14.9(6.2, 28.3)	97(96.2, 97.6)
ACC/AHA	29.3(19.4, 41)	81.2(79.5, 82.8)	5(3.2, 7.5)	97.1(96.3, 97.9)
Tanzania				
JNC7	11.3(6.2, 18.6)	96.2(95.6, 96.8)	7.7(4.2, 12.9)	97.5(97, 98.0)
ACC/AHA	42.6(33.4, 52.2)	76.9(75.6, 78.2)	4.9(3.7, 6.4)	98(97.4, 98.4)

## Discussion

This study reported a higher risk of stillbirth among pregnant women who reached the revised ACC/AHA criteria for hypertension, compared to normotensive counterparts. The risk of both stillbirth and preterm birth was marked when hypertension was defined using JNC7 criteria.

Previous observational studies in pregnant women with chronic hypertension have reported that blood pressures within the ranges of 120–129/80–89 mmHg, as opposed to ≥ 140/90 mmHg, were associated with lower risks of preterm birth, stillbirth, and other adverse perinatal outcomes including superimposed preeclampsia as compared to normotensive women [10–14]. Similarly, a multi-country trial from India, Mozambique, and Pakistan, reported the ACC/AHA defined stage 2 hypertension to be associated with adverse pregnancy outcomes as compared to normal blood pressure [15]. A meta-analysis of 13 studies involving 514,632 hypertensive pregnant women also revealed that the risk of preterm birth was 1.5 times higher with an ACC/AHA threshold above sBP 130 mmHg or dBP 80 mmHg (corresponding to stage 1 and 2 HTN) and 2 times higher with JNC7-defined hypertension. In the same study, the pooled risk for stillbirths was 1.6 and 3.5 using the ACC/AHA and JNC-7 criteria respectively. Consistent with our results, both thresholds have previously shown low sensitivity and other diagnostic capabilities in predicting stillbirths and preterm births. These outcomes depend on factors beyond hypertension, such as genetics, environmental influences, obstetric history, infections, and other complications, which cannot be ruled out by normal blood pressure measurements alone [16].

The ACC/AHA threshold identified an additional 1,437 (15.2%) pregnant women with hypertension in our cohort. We observed that examining every 43 women for ACC/AHA defined hypertension, could help detect one stillbirth outcome. Previously, Bello et al. reported that the reclassification of women as per the revised ACC/AHA criteria resulted in a 3.8% improvement in appropriate fetal/neonatal adverse outcome risk classification and a 20.8% improvement in the appropriate identification of future preeclampsia [17].

Hypertension in pregnancy is generally managed with serial blood pressure measurement, anti-hypertensive medication as needed, growth ultrasounds, and antenatal fetal testing to reduce the risk of superimposed preeclampsia [2]. However, optimal treatment thresholds and therapeutic goals can vary, depending on the balance between preventing maternal hypertensive complications and fetal adverse effects. The ACOG recommends initiation of anti-hypertensives at ≥ 160/110 mm Hg in cases of preeclampsia or chronic hypertension whereas other hypertension societies suggest treatment at ≥ 140/90 mm Hg to prevent severe hypertension [18]. Current guidelines suggest that a new diagnosis of elevated or stage 1 hypertension in a pregnant woman would not require initiation of anti-hypertensive medication, this is important in the case of LMICs where poor adherence to antenatal care (ANC) follow-up could lead to undetected progression of hypertension, increasing the stillbirth and preterm birth risk [18].

Previous randomized trials have reported that a treatment goal of SBP < 130 mm Hg accompanied by fetal growth monitoring is optimal for preventing adverse outcomes including stillbirths [19, 20, 21]. Thus, the identification of a moderate-risk population could help intensify clinical surveillance in these previously overlooked women. The costs of labelling a large proportion of the pregnant population as hypertensive could be balanced by efficient management of adverse perinatal outcomes and consequent healthcare costs. Thus, lowering thresholds enable timely blood pressure control and prolongation of pregnancies, particularly in high-risk cases with advanced maternal age, multiple gestations, and pre-existing health issues requiring aggressive management. It also facilitates targeted pregnancy-focused education for future risk reduction.

The strengths of our study include enrolling a large, community-based sample of pregnant women from three LMICs across South Asia and sub-Saharan Africa which have one of the highest burden of hypertensive pregnant women, and adverse pregnancy outcomes such as stillbirths and preterm births. We recorded blood pressure measurements at multiple time points during gestation using standardized procedures and pregnancy-validated devices. However, our study had limitations. Women with stage 2 hypertension were referred to facilities for antihypertensive treatment and facility care which could have affected the strength of association between lower levels of blood pressure and adverse outcomes. We did not adjust the risk ratios for factors such as maternal nutrition status, medication use, and obstetric history which affect blood pressure changes during pregnancy and the risk of adverse birth outcomes.

## Conclusion

The findings of this study suggest that revised ACC/AHA thresholds of elevated blood pressure (sBP 120–129 and dBP < 80), stage 1 hypertension (sBP 130–139 or dBP 80–89, or both), and stage 2 hypertension (sBP ≥ 140 or dBP ≥ 90, or both) were associated with an increased risk of stillbirths in pregnant women. The adoption of 2017 ACC/AHA hypertension criteria in pregnant women can help identify a moderate-risk population in need of clinical surveillance to permit timely control of blood pressure and avoidance of adverse outcomes.

### Electronic supplementary material

Below is the link to the electronic supplementary material.

Supplementary Material 1

## Data Availability

The datasets used and/or analyzed during the current study are available from the corresponding author on reasonable request.
