# Supplementary material for: Does the implementation of revised American College of Cardiology and American Heart Association (ACC/AHA) guidelines improve the identification of stillbirths and preterm births in hypertensive pregnancies: a population-based cohort study from South Asia and sub-Saharan Africa
Source: BMC Pregnancy Childbirth. 2024 Jun 29;24:451. doi: 10.1186/s12884-024-06637-2 (PMC11218258; doi:10.1186/s12884-024-06637-2)
Supplement: Supplementary file 1 — Supplementary Material 1 [file 12884_2024_6637_MOESM1_ESM.docx]

**Supplementary Table: S1 Blood pressure readings at antenatal care visits.**

| Baseline Characteristics | Bangladesh | Karachi | Pemba | Total |
| --- | --- | --- | --- | --- |
|  | **N=3,000**  **n (%)** | **N=2,500**  **n (%)** | **N=4,501**  **n (%)** | **N=10,001**  **n (%)** |
| Hypertension status at ANC 1 ℇ | n= 2,831 | n= 2,205 | n= 4,155 | n= 9,191 |
| Normal BP | 2,689 (95.0%) | 1,833 (83.1%) | 2,911 (70.1%) | 7,433 (80.9%) |
| Elevated BP | 44 (1.6%) | 151 (6.8%) | 637 (15.3%) | 832 (9.1%) |
| Stage 1 hypertension | 93 (3.3%) | 202 (9.2%) | 529 (12.7%) | 824 (9.0%) |
| Stage 2 hypertension | 5 (0.2%) | 19 (0.9%) | 78 (1.9%) | 102 (1.1%) |
|  |  |  |  |  |
| Hypertension status at ANC 2 ℇ | n= 2,748 | n= 2,118 | n= 4,029 | n= 8,895 |
| Normal BP | 2,551 (92.8%) | 1,676 (79.1%) | 2,863 (71.1%) | 7,090 (79.7%) |
| Elevated BP | 43 (1.6%) | 153 (7.2%) | 568 (14.1%) | 764 (8.6%) |
| Stage 1 hypertension | 140 (5.1%) | 260 (12.3%) | 492 (12.2%) | 892 (10.0%) |
| Stage 2 hypertension | 14 (0.5%) | 29 (1.4%) | 106 (2.6%) | 149 (1.7%) |

**Note:**
ℇ Hypertension Ranges For ANC 1 and ANC 2
 Normal BP (sBP <120 mm Hg and dBP <80 mm Hg)
 Elevated BP (sBP 120–129 mm Hg and dBP <80 mm Hg)
 Stage 1 hypertension (sBP 130–139 mm Hg or dBP 80–89 mm Hg, or both)
 Stage 2 hypertension (sBP >=140 mm Hg or >=dBP 90 mm Hg, or both)
